# Staining of Tubercle Bacilli

**Published:** 1888-06

**Authors:** T. E. Nott

**Affiliations:** Chattanooga, Tenn.


### STAINING OF TUBERCLE BACILLI.

BY T. E. NOTT, M. D., CHATTANOOGA, TENN.

Since the demonstration by Koch, some years ago, of the constant presence of a specific bacillus in the sputa of tuberculous subjects, we have passed through a series of slightly differing processes for its detection, from the decidedly complicated method first used by its eminent discoverer to the comparatively simple process most commonly employed to-day. In most works on pathology and microscopic investigation, the subject is fully treated, though perhaps not with a clearness and conciseness suited to the needs of the student and practitioner unused to pathological manipulations. It is hoped that this paper may present, in a short and condensed form, a satisfactory and easily understood method of staining these bacilli, giving the process step by step without any theorizing as to the different phenomena which arise. Of course nothing original is claimed, but the arrangement of the different formulæ which are thrown into prescription style instead of the usual "so many parts," which necessitates fresh calculation each time a specimen is stained. The first and one of the most absolute requisites is perfect cleanliness of instruments, vessels and water; the latter is preferably distilled; if this is not practicable it should at least be filtered. The apparatus staining fluids and reagents necessary are as follows:

Three or four deep watch-glasses, two needles in handles, delicate forceps, spirit lamp, slides and cover-glasses. To make fluids, there should be ℥iv absolute alcohol, ℥i nitric acid, ℥ii pure aniline oil, ℥i fuchsin. To contain the sputa, a small, wide-mouthed bottle is best; the ordinary one-eighth of morphia vial answers the purpose. Then proceed to make the following solutions :

SOLUTION A.

℞. Aniline Oil .......................... ʒi

Distilled Water .......................... ℥iii

M. and filter.

SOLUTION B.

℞. Alcohol .......................... ʒi

Fuchsin .......................... grs. xxx

M.

SOLUTION C.

Mix solutions A and B to make solution C.

Solution C is a "stock" preparation, and if the light be excluded will remain active for months.

SOLUTION D.

℞. Nitric Acid .......................... ʒi

Alcohol .......................... ℥iv

M.

Three watch-glasses are then placed in line upon a clean, fresh blotting-pad, the first to be filled with solution C, the second with solution D, while the third contains pure alcohol. A small portion of the suspected sputa is now placed by means of the needles upon a cover-glass, which has been cleansed with alcohol and afterwards thoroughly dried and polished with an old silk handkerchief. A second cover-glass is placed on the drop of sputa; they are laid upon the blotting-pad, and gentle pressure made upon them, so that a thin layer of sputa is formed between the two glasses; the excess around the edges is carefully removed with a handkerchief, and by a sliding motion the cover-glasses are separated, each one having a thin film of sputa on one side. They are laid *sputa side up* upon the blotting-pad. Then with the forceps, holding one of the glasses lightly by the edge, it is passed backward and forward two or three times through the flame of a spirit lamp with a motion about as rapid

"as that of cutting bread." By this the film of sputa is dried and fixed to the cover-glass.

It is then to be floated, sputa side down, in the first watch-glass; now lift the watch-glass carefully with forceps and pass backwards and forwards through the flame, not allowing the fluid to boil, but applying sufficient heat to cause thick vapors to rise. This process should be continued for about two minutes; then the watch-glass should be replaced upon the table and the specimen allowed to remain in the solution some five or six minutes after removing from the flame.

The cover-glass should now be lifted from the fluid and allowed to dry; a good way to effect this is to lean it against a large cork placed upon the pad. After having become dry, the film of sputa is seen to be colored intensely pink, nuclei, protoplasm and all micro-organisms present being stained by the fuchsin. It is then placed in the second watch-glass and allowed to remain simply immersed there for two or three minutes. The object of this is as follows: the acid removes from the protoplasm, micro-organisms, etc., the pink staining of the fuchsin, but does not affect the tubercle bacilli, allowing them, should they be present, to stand out in bold relief. This characteristic of resistance to acids is distinctive of the tubercle bacilli, with the exception of the leprosy bacillus; practically, however, we may regard the rule as absolute. Therefore, after having submitted one preparation to the action of the acid, we find, upon examination, pink-stained bacilli; we are justified in pronouncing them bacilli of tuberculosis.

From solution D the cover-glass preparation is transferred to the third watch-glass after thorough washing in the alcohol; it should be dried, a drop of glycerine placed upon a slide, the cover-glass upon the drop, *sputa side down*. Now, under a power of from 350 to 400 diameters, with a good light, the tubercle bacilli, should they be present, there may be seen tiny, hair-like rods stained deep pink by the fuchsin.

